# Intrapulmonary Percussive Ventilation as an Airway Clearance Technique during Venoarterial Extracorporeal Life Support in an Infant with Pertussis

**DOI:** 10.3389/fped.2017.00099

**Published:** 2017-04-27

**Authors:** Conrad Krawiec, Ken Ballinger, E. Scott Halstead

**Affiliations:** ^1^Department of Pediatrics, Division of Pediatric Critical Care Medicine, Penn State University College of Medicine, Hershey, PA, USA; ^2^Penn State Health Respiratory Care and Pulmonary Diagnostics, Penn State Health Children’s Hospital, Hershey, PA, USA

**Keywords:** acute respiratory distress syndrome, extracorporeal membrane oxygenation, pediatrics, Bordetella pertussis, whooping cough

## Abstract

Initiation of extracorporeal life support (ECLS) is often followed by complete opacification of pulmonary parenchyma and volume loss. The optimal mechanical ventilator management and lung recruitment strategy of a pediatric patient requiring extracorporeal membrane oxygenation is not known. We present a case of a 4-week old infant who developed a severe pertussis infection requiring ECLS. The severity of his illness and pertussis infection-associated intraluminal bronchiole obstruction made medical management challenging. In addition to lung protection ventilator strategies and bronchoscopy, intrapulmonary percussive ventilation was initiated to facilitate lung recruitment. This was associated with precipitous incremental improvement in lung compliance and eventual liberation from venoarterial ECLS.

## Background

After initiation of extracorporeal life support (ECLS) and hemodynamic stabilization, an early transformation in pulmonary compliance often occurs and is typically accompanied by chest radiograph “whiteout,” a complete opacification of pulmonary parenchyma and volume loss. It is speculated to be secondary to an acute decrease in airway pressure and a pro-inflammatory response secondary to exposure of blood to foreign circuit components (1–3).

“Whiteout” of the lung provides a new challenge to the clinician managing a patient with severe lung disease requiring ECLS. Careful mechanical ventilator management and lung recruitment in addition to routine critical care are crucial. Particular attention to the ventilator settings must be undertaken to avoid additional lung injury, and with time gradual improvement in lung aeration may occur.

Patience, however, may be difficult as the length of time spent on ECLS is related to survival (4). The clinician and critical care team must eventually decide when and how to facilitate recruitment of the lungs to improve native pulmonary function once recovery starts. What is unclear is the optimal way to recruit the lungs without causing further injury. Extracorporeal Life Support Organization guidelines recommend that lung recruitment after the initial inflammatory stage has resolved with “sigh breaths” limited to 25–30 cmH_2_O for 1–2 min, but there are no data on its safety and efficacy in ECMO patients (5). Kamat et al. utilized flexible bronchoscopy in pediatric patients on ECLS to provide pulmonary toilet, improve atelectasis, and recruit the lungs with a reported radiographic improvement median of 4.5 days (6). Flexible bronchoscopy, however, in an ECLS patient requires clinical experts, careful planning, and coordination to avoid complications, thus potentially minimizing the ability to aggressively use this technique. Recently, Michaels et al. developed a protocol using high-frequency percussive ventilation to facilitate alveolar recruitment and to improve native pulmonary function for adults treated with ECLS demonstrating a reduced duration of ECMO support when compared to modern cohorts (7). In the pediatric population, Yehya et al. demonstrated an increase in ECLS-free days in children receiving a combination of high-frequency percussive ventilation and bronchoscopy therapy for airway clearance and recruitment (8). High-frequency percussive ventilation, however, is not readily available at all institutions and requires expert training and management skills.

We report a novel use of intrapulmonary percussive ventilation in a case of severe pertussis requiring venoarterial ECLS in a facility where a high-frequency percussive ventilator was not readily available.

## Introduction

The patient is a 4-week old, 3.9 kg, Hispanic male who was born premature at 35 weeks gestational age and was admitted to Penn State Health Children’s Hospital as a transfer from an outside hospital with a 2-day history of cough, rhinitis, and apneic episodes. He was initially admitted to our Pediatric Intermediate Medical Care Unit, where he was diagnosed with a presumed viral illness after a sepsis rule out. During his hospitalization, he was started on broad spectrum antibiotics and continued to have repeated episodes of apnea. He was ultimately transferred to the Pediatric Intensive Care Unit due to respiratory failure, and mechanical ventilation was initiated. We summarized the major events that occurred during this patient’s course in Table 1.

**Table 1:** **Summary of the time course of major events during extracorporeal life support (ECLS) course**.

Major events	Pediatric intensive care unit Day #	ECLS Day #
Initiation of invasive mechanical ventilation	0	–
Double exchange transfusion	1	–
ECLS cannulation	2	0
Bronchoscopy #1	12	10
Bronchoscopy #2	15	13
Bronchoscopy #3	18	16
Development of right pleural effusion	18	16
Placement of right chest tube	20	18
Development of air leak syndrome	21	19
Placement of peritoneal drain	21	19
Bronchoscopy #4	23	21
Bronchoscopy #5	27	25
Bronchoscopy #6	28	26
Bronchoscopy #7	30	28
Bronchoscopy #8 (final)	33	31
Initiation of intrapulmonary percussive ventilation	33	31
Decannulation from ECLS	46	44

**Figure 1:**
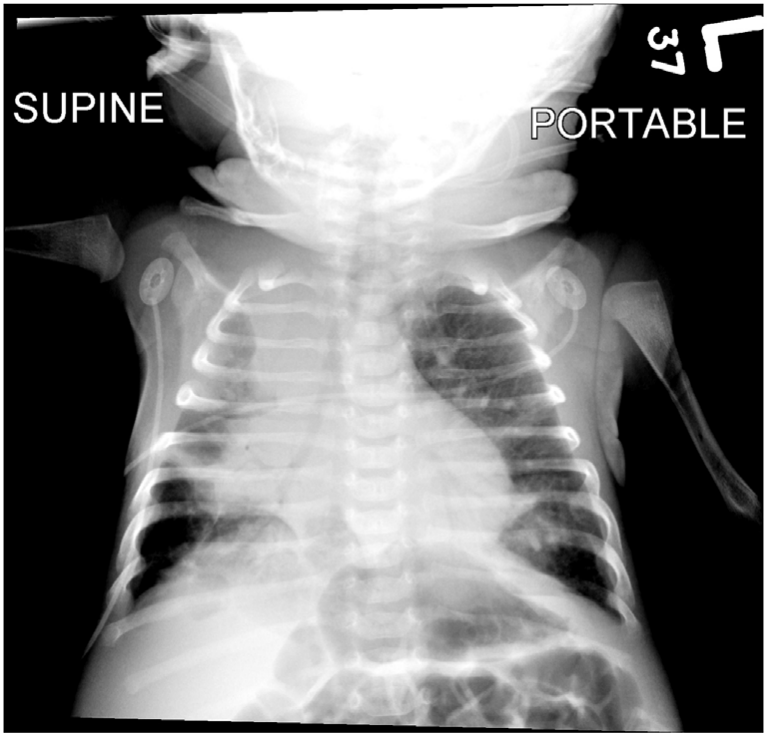
**A portable chest radiograph obtained earlier in the day demonstrating bilateral parenchymal opacities**.

The patient was ventilated using a Servo-i ventilator (Maquet, Germany) initially in the pressure-regulated volume control mode with settings of a tidal volume of 55 mL; a positive end-expiratory pressure (PEEP) of 5 cmH_2_O; a pressure support of 10 cmH_2_O; a respiratory rate (RR) of 26 breaths/min; and a fraction of inspired oxygen of 0.3. Peak inspiratory pressures (PIPs) were between 27 and 29 cmH_2_O. Initial diagnostic studies included a chest radiograph, which revealed dense opacification of the right upper lobe and patchy opacification of the left upper lobe and right middle lobe (Figure 1). He was also found to have a white blood cell count of 64.0 × 10^9^/L comprised of 60% lymphocytes. Nasopharyngeal polymerase chain reaction testing was positive for *Bordetella pertussis*.

Over the next 24 h, despite aggressive pulmonary toilet, initiation of methylprednisolone, a restrictive fluid strategy, and application of lung protective strategies, the patient’s clinical condition deteriorated with worsening lung compliance, decreasing from 1.96 mL/cmH_2_O (0.58 mL/cmH_2_O/kg) at admission to 0.86 mL/cmH_2_O (0.25 mL/cmH_2_O/kg) at 24 h. In addition, his WBC continued to rise (81.85 × 10^9^/L), and the patient also started to demonstrate elevated right ventricular pressures (50% systemic) *via* echocardiogram indicative of pulmonary hypertension.

As a means of leukoreduction, the patient underwent a 520 mL (150 mL/kg) double volume exchange transfusion using irradiated packed red blood cells reconstituted to an estimated hematocrit of 35%, in 20 mL aliquots. The WBC did decrease to 19.02 × 10^9^/L, but rose again to 33.9 × 10^9^/L within 6 h after the exchange. The patient’s PIPs continued to rise to 40 cmH_2_O with a plateau pressure of 38 cmH_2_O, and the decision to support the patient’s respiratory system with veno-venous extracorporeal life support (V-V ECLS) was made. An arterial blood gas prior to initiation was noted to have a pH of 7.451, PCO_2_ 61.8, and PaO_2_ 120.

After three relatively stable days on V-V ECLS with lung-protective ventilator management (PEEP 12 cmH_2_O, PIP 15 cmH_2_O, RR 12 bpm, and inspiratory time 1.4 s) and fluid restrictive strategies, the patient became hypotensive, poorly perfused, and demonstrated elevated central venous pressures above 20 mmHg, consistent with a pulmonary hypertensive crisis. The patient was then converted to veno-arterial (V-A) ECLS.

Immediately after placement on ECLS, the patient developed lung “whiteout” such that his tidal volume was reduced to only 1 mL total (0.3 mL/kg). To reestablish the patency of his airways, flexible bronchoscopy was started on ECLS Day 10 and performed multiple times. Lung recruitment was maintained with airway pressure release ventilation. On ECLS Day 16, the patient developed a right-sided pleural effusion, which was successfully drained *via* placement of a thoracostomy tube with subsequent improved recruitment of the lungs on ECLS Day 18. The patient then developed air leak syndrome on ECLS Day 19, which comprised of pneumomediastinum, pneumoperitoneum, and subcutaneous emphysema necessitating the placement of a peritoneal drain. The patient was placed on total lung rest *via* continuous positive airway pressure of 5 cmH_2_O. After the patient’s air leak had resolved we experienced difficulty in re-recruiting the lung. Despite repeated bronchoscopies (five times on different days) and various PEEP settings, only 2 mL/kg of tidal volume was being generated with pressure control 20; PEEP of 8 cmH_2_O. High-dose (4 mg/kg) methylprednisolone was initiated for possible late acute respiratory distress syndrome. Review of available bronchoalveolar lavage cultures obtained on ECLS #31 showed no evidence of any superbacterial infection and resolution of previously noted growth of yeast on ECLS Days 10 and 13.

**Figure 2:**
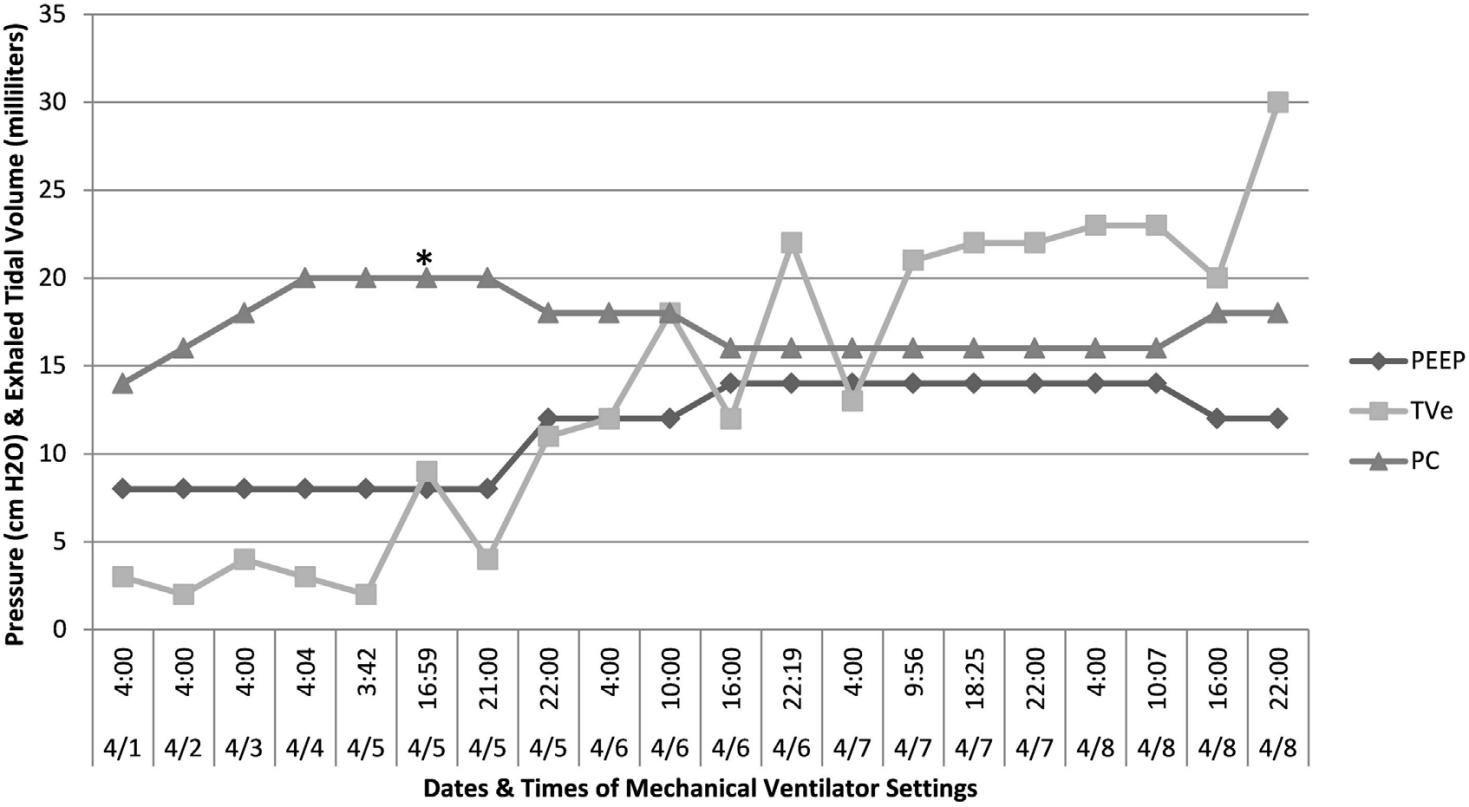
**Time course of initiation of IPV therapy and its effects on pulmonary compliance**. After initiation of IPV therapy and concomitant titration of PEEP, there was sustained recruitment of alveoli evidenced by an increase in the amount of exhaled tidal volume. *Intrapulmonary percussive ventilation therapy. IPV; PEEP, positive end-expiratory pressure; TVe, exhaled tidal volume; PC, pressure control setting.

We hypothesized that the repeated bronchoscopy and airway recruitment maneuvers were ineffective secondary to small airway plugging due to the continued presence of mucus and luminal debris. Therefore, on ECLS Day 31, to mobilize this mucus and debris we initiated intrapulmonary percussive ventilator (IPV) therapy. In our unit, we utilize the Percussionaire^®^ Corporation Model IPV-1C intrapulmonary percussive ventilation device. Prior to treatment, we ensured that the patient was sedated and paralyzed to avoid active expiration during the procedure. Typical treatments lasted from 15 to 20 min depending on the individual respiratory therapist. The initial settings were a driving pressure of 35 psi with verified chest wiggle during therapy. Since the patient was removed from the ventilator during the procedure, the percussive interval was limited to 15–30 s and was followed by manual ventilation breaths through the IPV machine with close monitoring of vital signs. Small boluses of sodium bicarbonate and *N*-acetylcysteine (alternated every 4 h) were instilled down the endotracheal tube a few minutes apart, while operating the IPV with nebulized normal saline and varying the frequency settings between “easy” and “hard.” Initial treatments were productive for moderate amounts of thick creamy secretions, with increased lung volumes noted. Each treatment was followed by manual sigh breaths with an extended inspiratory time and an incremental increase in ventilator PEEP to a maximum of 14 cmH_2_O, to facilitate and maintain lung recruitment (see Figures 1 and 2).

**Figure 3:**
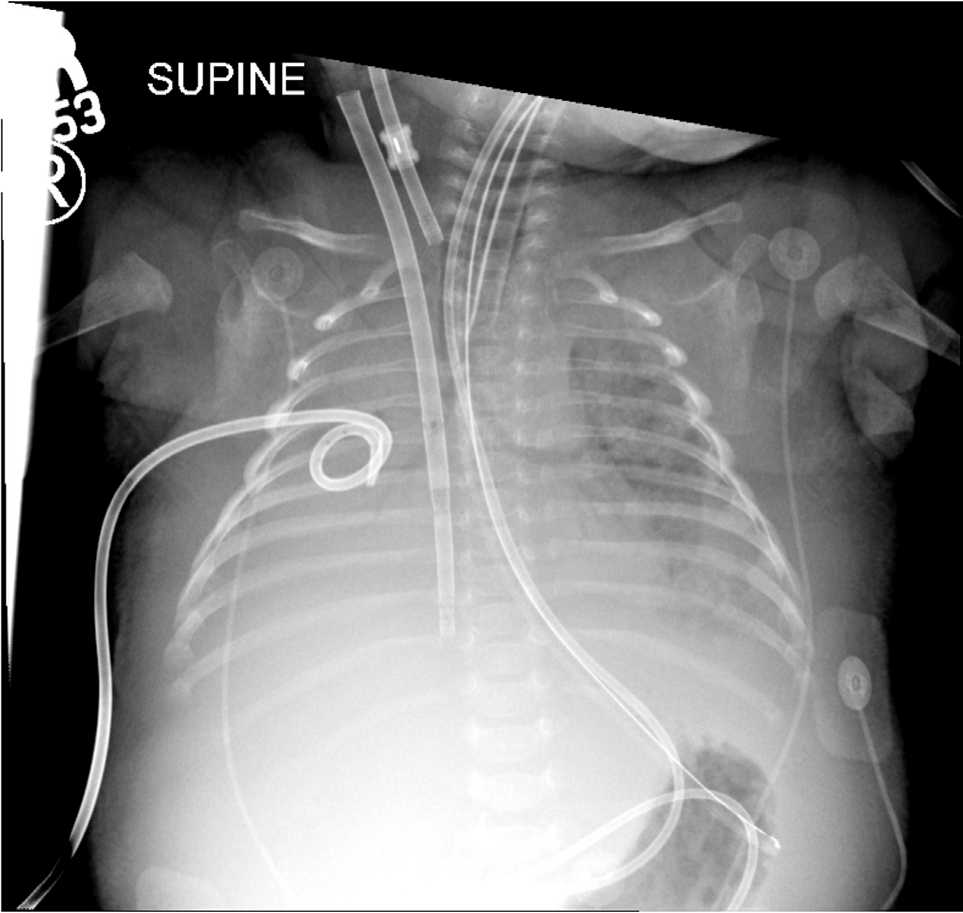
**Chest radiographs before initiation of intrapulmonary percussive ventilator therapy and 4 hours after bronchoscopy**. There was improvement of aeration of the left lung with persistent hazy opacifications in the right upper and middle lobes.

**Figure 4:**
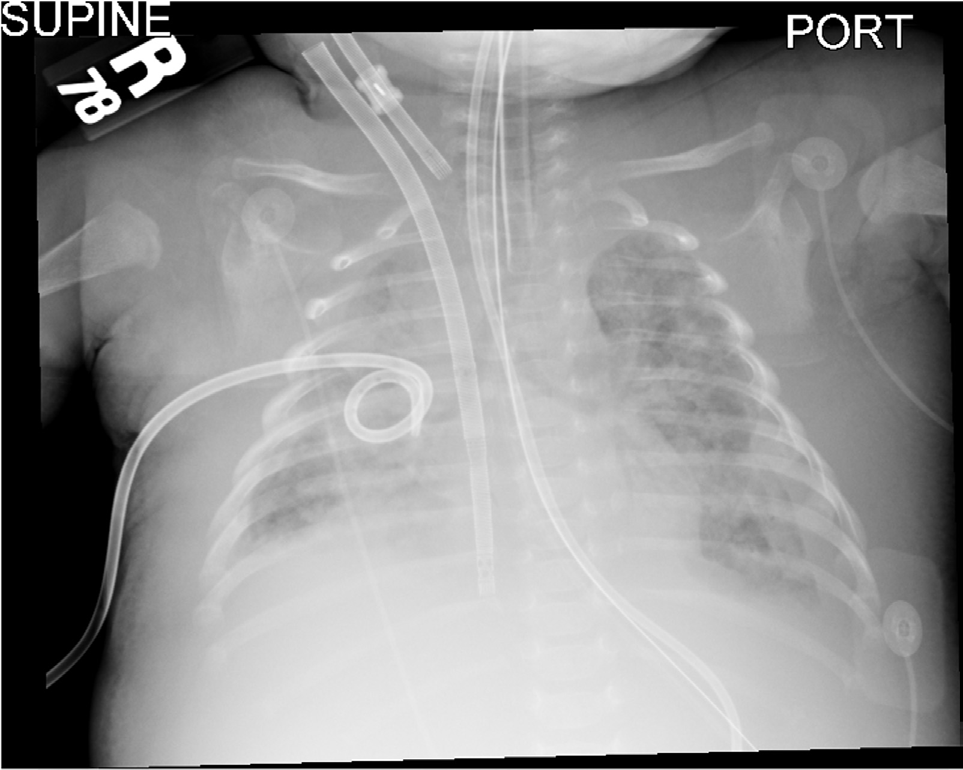
**Chest radiographs 6 h after initiation of intrapulmonary percussive ventilator therapy**. There was improvement of aeration of the left lung with persistent hazy opacifications in the right upper and middle lobes.

**Figure 5:**
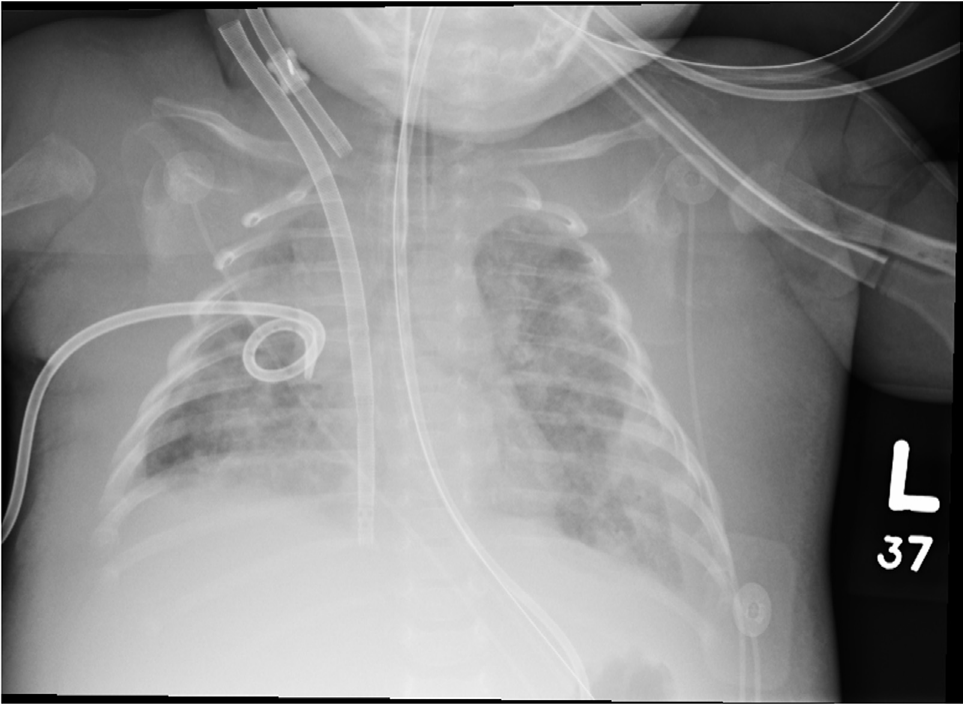
**Twelve hours after initiation of intrapulmonary percussive ventilator there was continued improved aeration of both lungs**.

At the onset of IPV therapy, the patient’s lungs were whited out on chest radiograph with low lung compliance and exhaled tidal volumes of 0.5 mL/kg (Figures 2 and 3). After initiation of therapy, tidal volumes and lung compliance incrementally improved, such that at Day 5, exhaled tidal volumes were 7 mL/kg and chest radiographs showed dramatically less infiltrative processes (Figures 4 and 5). Within 10 days, the patient was weaned off V-A ECLS to conventional ventilation. Bronchoscopy was no longer needed since the initiation of intrapulmonary percussive ventilation therapy.

Unfortunately, while the patient’s lung injury improved during his 44-day ECLS course and he survived to hospital discharge, the ECLS course was complicated by multiorgan dysfunction syndrome secondary to fungemia and sepsis, and he developed extensive hypoxic ischemic injury of the basal ganglia and thalamus.

## Discussion

Our patient had developed a severe pertussis infection that is associated with intraluminal bronchiole obstruction likely secondary to mucus (9, 10). Lung recruitment may have been limited by the inability to suction and clear mucus secretions, and therefore initially bronchoscopy was successfully recruited his lungs. However, bronchoscopy can be limited by expert operator availability and by the fact that it can only access second–third generation airways. An additional recruitment maneuver was needed to facilitate airway clearance as our standard of care was not effective. High-frequency percussive ventilation was considered, but not able to be obtained due to device availability.

Instead, we opted to employ intrapulmonary percussive ventilation, a method of airway clearance that can be performed by our respiratory therapists periodically. Intrapulmonary percussive ventilation utilizes a spring-controlled venturi system (phasitron) powered by compressed gas. The venturi system slides back and forth in a percussive manner, delivering high frequency, low volume bursts of positive pressure. Using an asymmetric flow pattern (where expiratory flow is greater than inspiratory flow) and increasing the diameter of the airway, this device propels endobronchial secretions centrally and recruits obstructed bronchi and alveoli (11, 12). This device is currently utilized for various types of disease processes including chronic obstructive pulmonary disease, children with cystic fibrosis, and it has been used in patients with neuromuscular weakness, obesity, and tracheostomies who were subject to atelectasis (11, 13–16). In addition, it has also been used acutely to treat severe pneumonia requiring airway mucus clearance and recruitment of the affected lung superimposed on conventional mechanical ventilation (17, 18). Because of its applications in other disease processes, its ability to mobilize secretions and similarities to high-frequency percussive ventilation, we felt that this device would be useful in this patient’s disease process (19).

After application of this device, over the next 7 days, the patient’s oxygenation and pulmonary compliance started to improve. This therapy allowed secretion clearance and with a slow upward titration of PEEP, the lungs were able to be recruited. To date, there are no data utilizing intrapulmonary percussive therapy in this fashion in patients requiring ECLS. Therefore, it may provide a novel way to provide airway secretion clearance and allow recruitment of healing alveoli. In addition, because of the lack of widespread availability and use, this form of lung recruitment can be considered instead of high-frequency percussive ventilation as the only disadvantage is the coordination of conventional ventilation, high-frequency oscillation, and percussion provided by this device (19).

In a patient on ECLS, however, this device is not without risk. During treatments, there were reports of blood tinged secretions that resolved when *N*-acetylcysteine was discontinued. There were reports of hemorrhage from the cannula site that resolved when this treatment was discontinued for 24 h. Finally, the patient also developed a small pneumothorax of the left lung that resolved spontaneously within 48 h after discontinuation of this treatment.

## Concluding Remarks

In conclusion, when faced with a pulmonary disease that is associated with intraluminal bronchiole mucus obstruction this type of therapy should be considered in addition to lung protective strategies and bronchoscopy. Intrapulmonary percussive ventilation was applied with marked lung recruitment in a short amount of time, and it may be an option for aggressive airway clearance with bronchoscopy. It also may be a way to obtain the same type of potential benefit from high-frequency percussive ventilation in institutions that do not routinely use this type of ventilation.

## Ethics Statement

This case report was reviewed by our institutional review board (Human Subjects Protection Office, Penn State) and determined that the proposed activity (Study ID: STUDY0006593) does not meet the definition of human subject research as defined in 45 CFR 46.102(d) and/or (f). Verbal informed consent was obtained from the patient’s mother for publication of this case report, but despite multiple attempts, written informed consent was unable to be obtained. A copy of the attestation of verbal consent is available for review by the Editor-in-Chief.

## Author Contributions

The drafting of the manuscript was performed by CK and KB. Critical revision of the manuscript for important intellectual content was performed by SH.

## Conflict of Interest Statement

The authors declare that the research was conducted in the absence of any commercial or financial relationships that could be construed as a potential conflict of interest.
